# Pharmacological Treatment in the Management of Chronic Subdural Hematoma

**DOI:** 10.3389/fnagi.2021.684501

**Published:** 2021-07-01

**Authors:** Xing Wang, Jinlei Song, Qiang He, Chao You

**Affiliations:** ^1^Department of Neurosurgery, West China Hospital, Sichuan University, Chengdu, China; ^2^Department of Gastroenterology and Hepatology, West China Hospital, Sichuan University, Chengdu, China; ^3^Department of Neurosurgery, West China Brain Research Centre, Sichuan University, Chengdu, China

**Keywords:** chronic subdural hematoma, drug therapy, dexamethasone, tranexamic acid, network meta-analysis

## Abstract

**Background:** Several pharmacological treatments have been used to treat patients with chronic subdural hematoma (CSDH), although little is known about the comparative effectiveness of different classes of medication. We performed a Bayesian network meta-analysis to compare and rank the efficacy and safety of five drug regimens to determine the best treatment for this group of patients.

**Methods:** We systematically searched PubMed, Medline, clinicaltrials.gov, the Cochrane database, and Embase to identify relevant randomized clinical trials (RCTs) comparing drug treatments in adult patients with CSDH. A network meta-analysis was conducted using a Bayesian framework. Random- and fixed-effects models were used to pool the network results, and the preferred model was selected by comparing the deviance information criteria (DIC). Efficacy outcomes included recurrence requiring surgery, changes in hematoma volume, and a good recovery. The safety outcomes were treatment-related adverse events and all-cause mortality.

**Results:** In this Bayesian network meta-analysis, available data were obtained from 12 eligible trials, including 2,098 patients and 5 techniques. Compared to placebo, atorvastatin (RR: 0.45, 95% CrI: 0.24–0.81) and dexamethasone (RR: 0.38, 95% CrI: 0.22–0.63) were similarly effective in reducing recurrence requiring surgery by 55% and 62%, respectively. Dexamethasone (RR: 0.46, 95% CrI: 0.23–0.91) was more effective in reducing recurrence requiring surgery than goreisan. Additionally, atorvastatin reduced the hematoma volume to a greater extent than placebo (MD: −7.44, 95% CrI: −9.49 to −5.43) or goreisan (MD: −14.09, 95% CrI: −23.35 to −4.82). Moreover, tranexamic acid (MD: −12.07, 95% CrI: −21.68 to −2.29) reduced the hematoma volume to a greater extent than goreisan. No significant differences were detected between drugs and placebo with regard to a good recovery. In terms of safety, dexamethasone (RR: 1.96, 95% CrI: 1.20–3.28) increased the risk of mortality compared to placebo.

**Conclusion:** These findings suggest that dexamethasone is the best treatment to reduce recurrence and atorvastatin is the best treatment to reduce hematoma volume in patients with CSDH. However, clinicians should pay close attention to the elevated risk of all-cause mortality and potential adverse events caused by dexamethasone. Future well-designed RCTs with more participants are needed to verify these findings.

**Clinical Trial Registration:** http://osf.io/u9hqp.

## Introduction

Chronic subdural hematoma (CSDH) is one of the most common neurosurgical diseases and is more frequent in the elderly than in other populations. The prevalence of CSDH is approximately 13.1/100,000 persons, but reaches 127/100,000 persons among individuals who are ≥80 years old (Kudo et al., 1992; Balser et al., 2015; Brennan et al., 2017; Rauhala et al., 2020). Given the aging of the population, the prevalence of CSDH will likely rise in the future.

Treatment for CSDH can be either conservative or surgical depending on the general condition of patients, as well as their symptoms and hematoma volume (Santarius et al., 2009; Soleman et al., 2017). However, high-level evidence to guide CSDH treatment is currently lacking. Although surgery is still considered a straightforward and safe procedure in cases of neurological impairment, the recurrence rate is relatively high (Weigel et al., 2003; Liu et al., 2014; Peng and Zhu, 2016). Surgery tends to be contraindicated in the elderly patients due to preexisting co-morbidities. Therefore, the identification of a safe and effective non-surgical treatment of CSDH is important (Bender and Christoff, 1974; Holl et al., 2018; Huang et al., 2020). In line with this, pharmacological treatment such as dexamethasone, atorvastatin, and tranexamic acid as an adjuvant or alternative therapy to surgery may be a worthwhile avenue to explore (Berghauser Pont et al., 2012b; Anker-Moller et al., 2017; Qiu et al., 2017). Standard guidelines on the first rank drug treatment of CSDH are currently lacking, which makes it challenging for clinicians and patients to decide on a medication. Therefore, it is necessary to perform a network meta-analysis to investigate the relative effect of different treatment regimens and provide a more comprehensive analysis of the currently available evidence.

We conducted a Bayesian network meta-analysis to compare and rank the efficacy and safety of the pharmacological treatments of CSDH that have been applied in clinical practice. We also performed a comprehensive ranking of various medications to determine which one can efficiently and safely reduce recurrence of CSDH.

## Materials and Methods

### Protocol and Guidance

The methods of reporting the present meta-analysis complied with the PRISMA-Network meta-analysis (PRISMA-NMA) guidelines (Hutton et al., 2015). This study was registered with Open Science Framework database (http://osf.io/u9hqp).

### Selection Criteria

Eligible studies satisfied the following criteria relating to participants, interventions, comparators, outcomes, and study design: Adult patients (age >18 years old) with CSDH regardless of severity; administration of any pharmaceutical treatment; no limitations on the dose and method of administration of drugs; placebo or another single drug; efficacy outcomes including recurrence requiring surgery, changes in hematoma volume, and good recovery [defined as Markwalder Grading Scale (MGS) = 0 (Morgan et al., 2013); Glasgow Coma Scale (GCS) score = 15 (Reith et al., 2016); modified Rankin Scale (mRS) score = 0–2 (Quinn et al., 2009); Glasgow Outcome Scale (GOS) score = 5 (Jennett et al., 1981)]; safety outcomes including mortality from any cause and adverse events (we extracted data on treatment-related adverse events if available; otherwise, the adverse events that were more frequently reported in the intervention group were extracted); and randomized controlled trials.

### Search Strategy

We searched Ovid Embase, Pubmed, Cochrane Central Register of Controlled Trials (CENTRAL), Ovid Medline, and clinicaltrials.gov for relevant manuscripts written in any language from inception to March 8, 2021. In cases where non-English articles were identified, we sought assistance from a professor of linguistics from our University or online translation software. We also conducted a thorough search of the references of the selected studies and several published systematic reviews on the same field to identify additional studies. Search strategy was presented in Supplementary Table 1.

### Selection Process

The study selection process stringently followed the PRISMA-NMA guidelines. After deleting duplicates, two reviewers manually filtered publications that were found to be ineligible based on the screening of the titles and/or abstracts. Subsequently, full-text articles were excluded based on the aforementioned criteria. Two reviewers independently completed this procedure together. In the case of disagreement, a third independent reviewer made the final decision.

### Data Extraction

Data associated with the following items were extracted into a standardized form: (1) study characteristics, including the first author, publication year, geographical location, and follow-up period; (2) patient characteristics, including mean age and proportion of males; and (3) treatment characteristics, including the type of medication, dosage, and duration of treatment.

Two reviewers independently extracted data from the included trials using a standardized extraction form. In the case of deficient data, we contacted the corresponding authors of the articles for clarification. Disagreements were resolved by consensus or determined by a third independent reviewer.

### Evaluation of Risk of Bias and Quality of Evidence

Risk of bias was evaluated for all of the included trials using the Cochrane Collaboration Risk of Bias tool according to the following domains: incomplete outcome data, random sequence generation, allocation concealment, blinding of study participants, selective reporting, blinding of outcome assessment, and other potential sources of bias (Higgins et al., 2011). Each domain was rated as either low, unclear, or high risk of bias. A trial was rated as low risk of bias overall if all domains were found to have a low risk of bias; otherwise, the trial was rated as a high risk of bias overall. We contacted the original study investigators for more information if necessary.

The quality of evidence for the outcomes was assessed using a framework developed by the Grading of Recommendations Assessment, Development, and Evaluation (GRADE) working group for rating the quality of effect estimates (Guyatt et al., 2008). Five domains were assessed, including limitations in design, publication bias, inconsistency, imprecision, and indirectness, and the synthesized quality of evidence for each outcome was rated as “high,” “moderate,” “low,” or “very low.”

### Statistical Analysis

Bayesian network meta-analysis was conducted using the R software package gemtc. A consistency model was established to combine direct and indirect comparisons. Random- and fixed-effects models were used to pool the network results and the preferred model by comparing the deviance information criteria (DIC) (Spiegelhalter et al., 2002; McGavock et al., 2020). The comparative efficacy and safety of any two treatment regimens was modeled such that each drug was relative to the other, and the point estimates [relative risks (RRs), or mean differences (MDs)] and the relevant 95% credible intervals (CrIs) were then obtained from the model. We also devised a Markov chain Monte Carlo model with 30,000 simulated draws after a burn-in with 10,000 iterations. Network geometry graphs were used to represent all available direct comparisons between the interventions for each efficacy and safety outcome. A ranking probabilities graph was used to visually rank the hierarchy of various types of medication in the network meta-analysis. Inconsistency was assessed by comparing the direct and indirect evidence using a node-splitting approach if relevant head-to-head trials were available. We assessed global statistical heterogeneity across all comparisons using the *I*^2^ statistic, where <25% was considered low, 25–50% was considered moderate, and >50% was considered high.

We calculated the RRs with 95% CrIs for dichotomous outcomes, and MDs with 95% CrIs for continuous outcomes. In case of continuous variables that provided incomplete or inexhaustive results, we used the formula suggested by the Cochrane Handbook for Systematic Reviews of Interventions. To ensure the robustness of the findings, sensitivity analysis was conducted by excluding trials that used drugs as an alternative to surgical treatment.

Two-sided *p*-values <0.05 were considered to be statistically significant. All analyses were performed using R software (release version 4.0.3) and RevMan software (5.4.1; The Cochrane Collaboration).

## Results

### Eligible Studies and Study Characteristics

**Figure 1:**
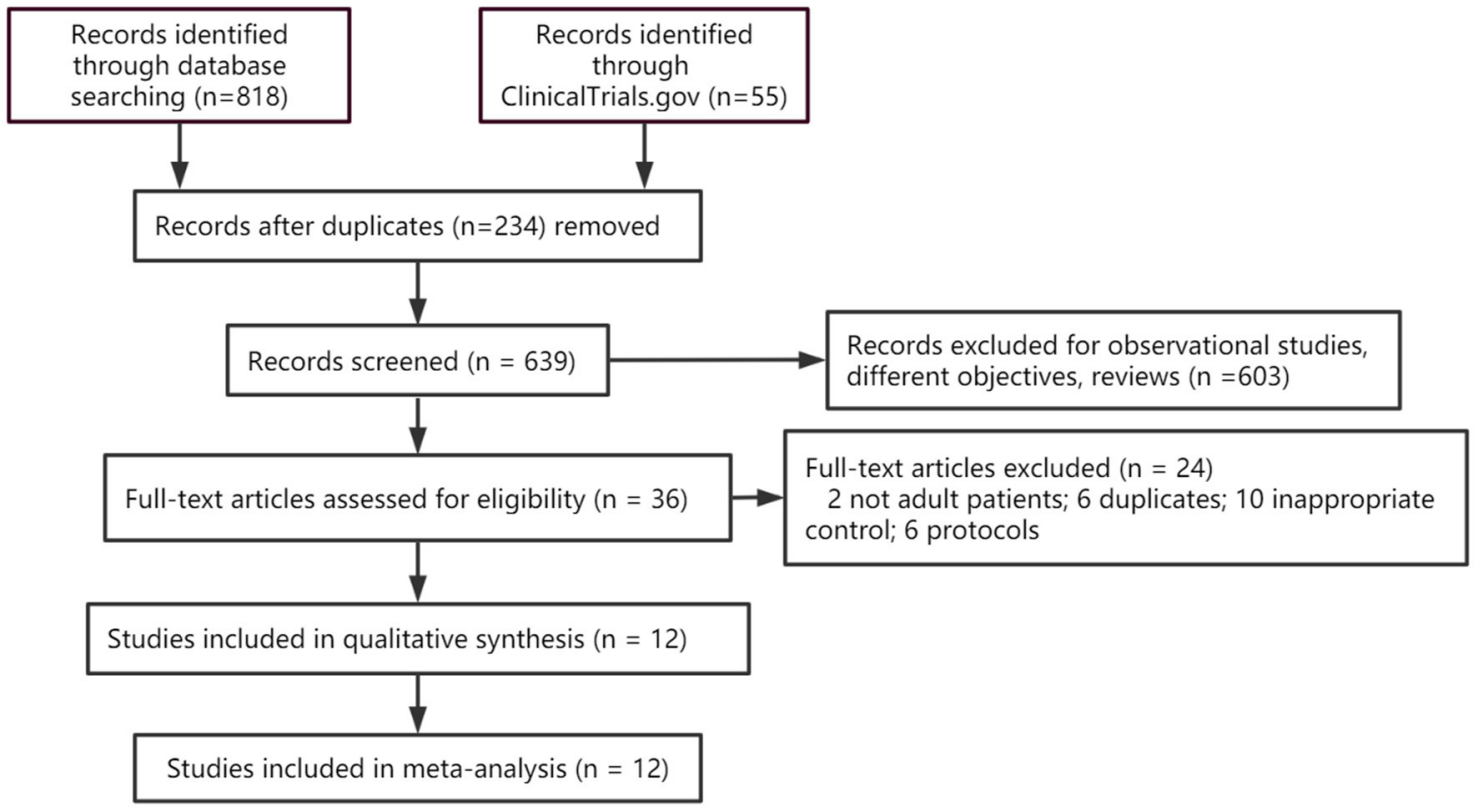
PRISMA flow diagram showing study selection. RCT, Randomized controlled trials.

The database search yielded 873 articles. Finally, 12 controlled trials were deemed eligible for inclusion in the Bayesian meta-analysis (Poulsen et al., 2014; Chan et al., 2015; Prud'homme et al., 2016; Jiang et al., 2018; Katayama et al., 2018; Workewych et al., 2018; Hutchinson et al., 2020; Mebberson et al., 2020; Wan et al., 2020; Yamada and Natori, 2020; Yang et al., 2020; Fujisawa et al., 2021). The PRISMA flow chart showing the publication selection process and a list of studies with reasons for exclusion are provided in Figure 1.

The study characteristics are presented in Table 1. The eligible trials were published from 2014 to 2021, with population sizes ranging from 20 to 748 participants. One trial assessed perindopril (Poulsen et al., 2014); two trial assessed atorvastatin (Jiang et al., 2018; Yang et al., 2020); two trials assessed goreisan (Katayama et al., 2018; Fujisawa et al., 2021); three trials assessed tranexamic acid (Workewych et al., 2018; Wan et al., 2020; Yamada and Natori, 2020); and four trials assessed dexamethasone (Chan et al., 2015; Prud'homme et al., 2016; Hutchinson et al., 2020; Mebberson et al., 2020). One of the included randomized clinical trials (RCTs) was a three-arm trial (Yamada and Natori, 2020). The majority of trials mainly enrolled elderly male patients. The proportion of males in the control group ranged from 46 to 100%, and the mean age of the participants in the control group of each trial ranged from 60.2 to 78.8 years. A network plot of eligible comparisons is presented in Figure 2.

**Table 1:** Characteristics of studies included in the systematic review and network meta-analysis.

**Study**	**Registration**	**Country (centers)**	**Age (% male)^a^**	**No. of patients**	**Intervention**	**Control**	**Type of surgery**	**Primary outcomes**	**Duration of follow-up**
Poulsen et al., 2014	NCT00915928	Denmark	70.5 (77.3%)	47	Perindopril 5 mg	Placebo	Burr hole surgery	Recurrence requiring surgical intervention	6 weeks
Jiang et al., 2018^#^	NCT02024373	China	67.0 (90.0%)^b^	196	Atorvastatin 20 mg	Placebo	NA	Changes in HV	8 weeks
Yang et al., 2020	NA	China	60.2 (53.6%)	58	Atorvastatin 20 mg	Placebo	Not mentioned	Hematoma elimination	24 weeks
Katayama et al., 2018	UMIN000015970	Japan	75.8 (76.1%)	180	Goreisan 7.5 g	Placebo	Burr hole surgery	Recurrence requiring surgical intervention	12 weeks
Fujisawa et al., 2021	UMIN000010006	Japan	74 (72.1%)^b^	208	Goreisan 7.5 g	Placebo	Burr hole surgery	Symptomatic recurrence	3 months
Workewych et al., 2018	NCT03280212	Canada	70.9 (46 %)	24	TXA 500 mg	Placebo	Burr-hole surgery or mini-craniotomy	Changes in HV	8 weeks
Yamada and Natori, 2020	NA	Japan	78.8 (62.5%)	232	TXA 750 mg; Goreisan 7.5 g	Placebo	Burr hole surgery	Recurrence requiring surgical intervention	3 months
Wan et al., 2020	NA	Singapore	69.6 (73.5%)	90	TXA 1,000 mg	Placebo	Burr-hole surgery or mini-craniotomy	Recurrence requiring surgical intervention	24 weeks
Prud'homme et al., 2016^#^	NCT02362321	Canada	69.4 (100%)	20	Dexamethasone 12 mg	Placebo	NA	Rate of success of conservative management	6 months
Mebberson et al., 2020	ACTRN12613000175774	Australia	75.1 (79.0%)	47	Dexamethasone 12 mg	Placebo	Burr-hole surgery or craniotomy	Mortality, and recurrence requiring surgical intervention	6 months
Hutchinson et al., 2020	ISRCTN80782810	UK	74.3 (76.7%)	748	Dexamethasone 12 mg	Placebo	Burr hole surgery	Modified Rankin scale 0–3	6 months
Chan et al., 2015	NA	Hong Kong	71.9 (73.0%)	248	Dexamethasone 24 mg and Surgery	Surgery	Burr hole surgery	Recurrence requiring surgical intervention	6 months

*NA, not available; HV, hematoma volume; TXA, tranexamic acid; UK, United Kingdom*.

a *These data were extracted from control group*.

b *Age was presented in median*.

# *Drug was administered as an alternative to surgery*.

**Figure 2:**
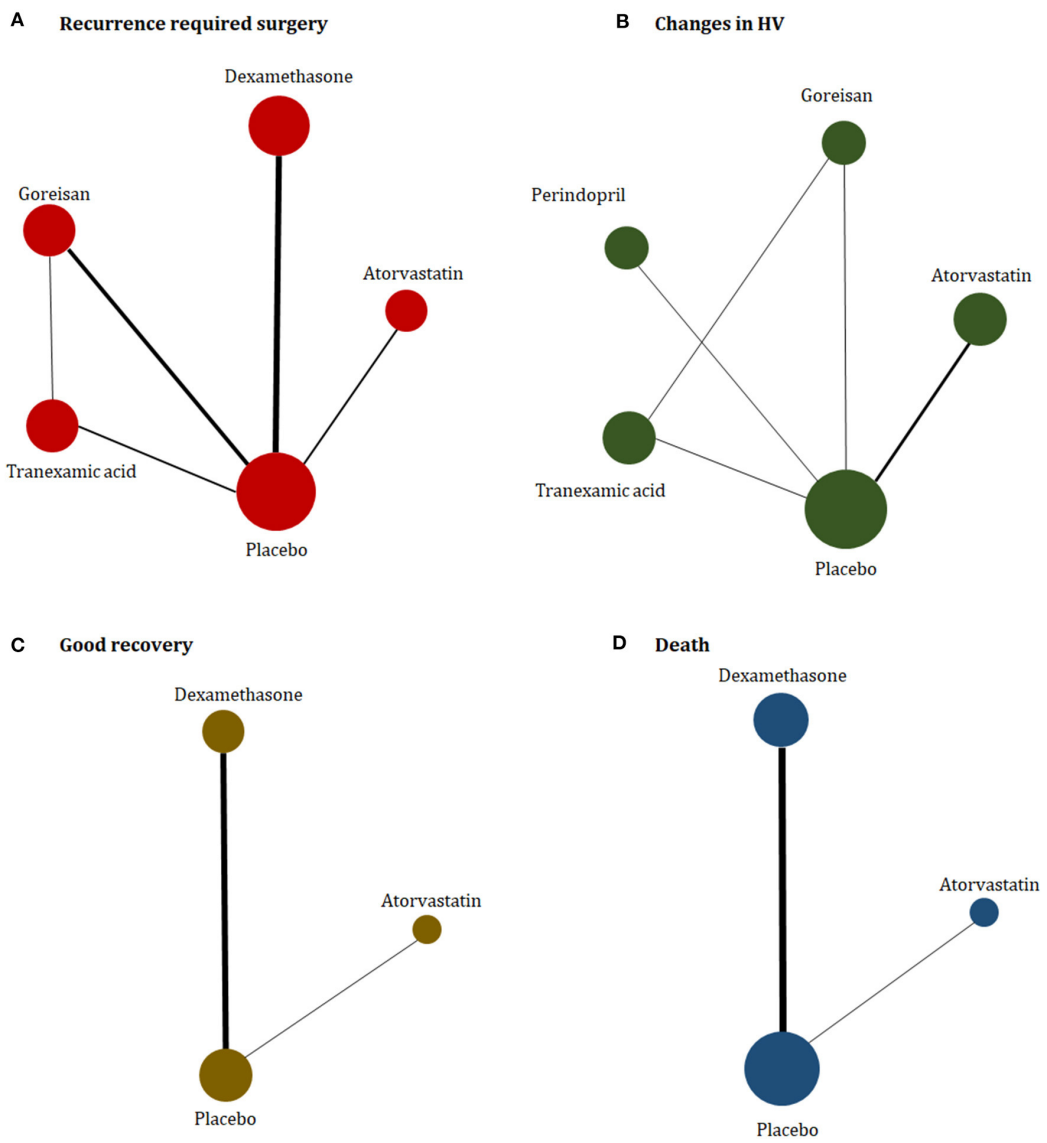
Network plot of **(A)** recurrence required for surgery, **(B)** changes in hematoma volume, **(C)** good recovery, and **(D)** death. The width of the lines is proportional to the number of studies comparing every pair of treatments, and the size of each circle is proportional to the number of participants.

### Risk of Bias

Three trials were regarded as having an overall low risk of bias, which demonstrated that the selected RCTs were of good quality; the remaining nine trials were found to have an overall high risk of bias. Most trials were rated as holding a high risk of bias due to unblinding of participants, personnel, and/or outcome assessment. Detailed information on the assessment of risk of bias for each trial is provided in Supplementary Figures 1, 2.

### Efficacy Outcomes

**Figure 3:**
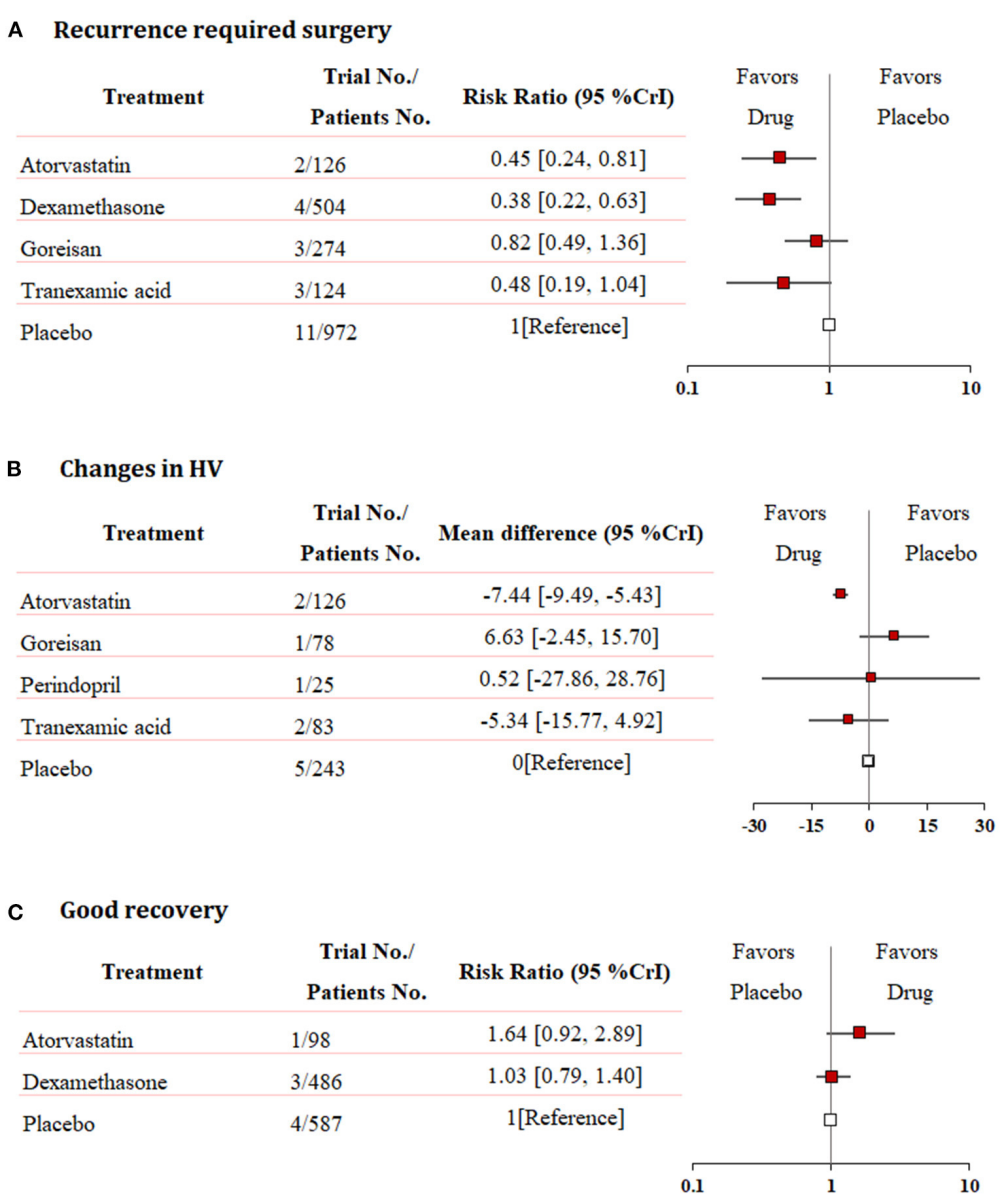
Network meta-analysis of efficacy outcomes. **(A)** Recurrence requiring surgery, **(B)** changes in hematoma volume, and **(C)** good recovery. Each medication is compared with placebo (reference). RR, relative risk; MD, mean difference; CrI, credible interval; HV, hematoma volume.

**Figure 4:**
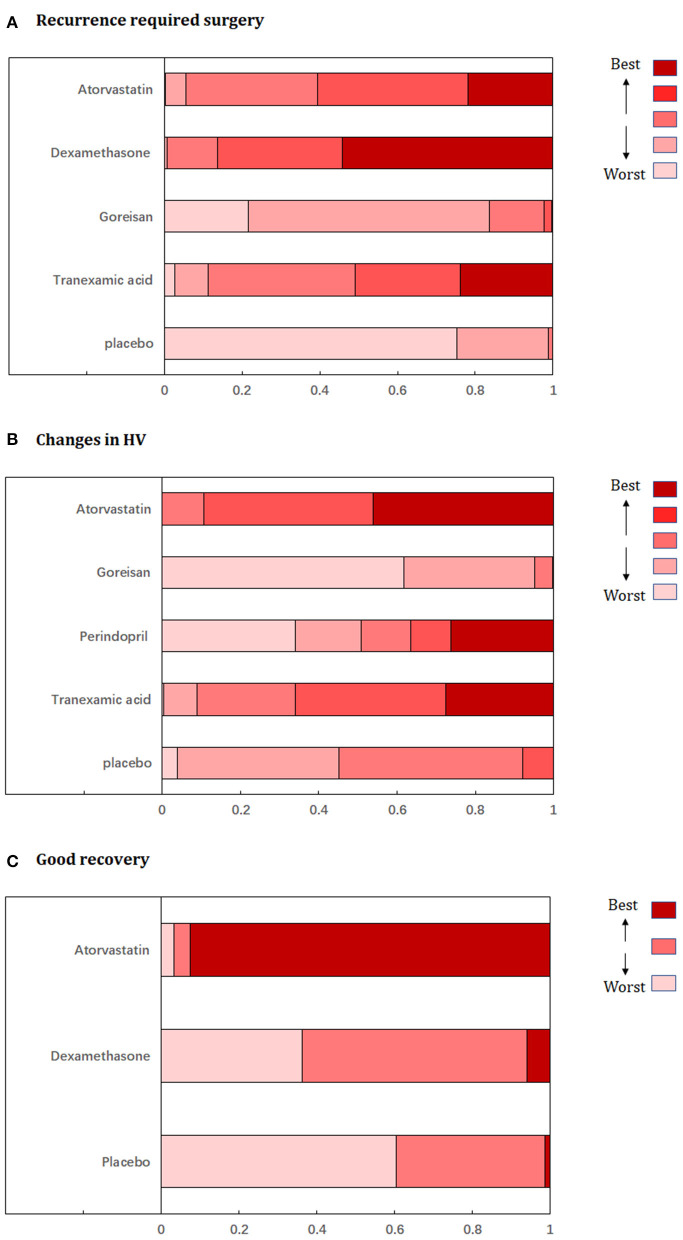
Ranking probabilities graph of each medication for **(A)** recurrence requiring surgery, **(B)** changes in hematoma volume, **(C)** good recovery. HV, hematoma volume.

Fixed-effects models yielded better DIC than random-effects models for all estimates except a good recovery (Supplementary Table 2). With regard to efficacy outcomes, recurrence requiring surgery was found in four treatment regimens involving 2,000 patients. According to the analysis, atorvastatin (RR: 0.45, 95% CrI: 0.24–0.81, Figure 3A) and dexamethasone (RR: 0.38, 95% CrI: 0.22–0.63, Figure 3A) decreased the risk of CSDH recurrence requiring surgery. Dexamethasone was more effective in reducing recurrence than goreisan (RR: 0.46, 95% CrI: 0.23–0.91). Dexamethasone had the highest probability of being ranked first for reducing recurrence, followed by atorvastatin, tranexamic acid (RR: 0.48, 95% CrI: 0.19–1.04, Figure 3A), and goreisan (RR: 0.82, 95% CrI: 0.49–1.36, Figure 3A). The ranking positions of each drug are presented in Figure 4A. Five randomized trials, including 555 patients, provided data on changes in hematoma volume. Overall, atorvastatin showed better efficacy than placebo in reducing the hematoma volume (MD: −7.44, 95% CrI: −9.49 to −5.43). We also found that atorvastatin (MD: −14.09, 95% CrI: −23.35 to −4.82) and tranexamic acid (MD: −12.07, 95% CrI: −21.68 to −2.29) reduced the hematoma volume to a great extent than goreisan. Atorvastatin had the highest probability of being ranked first for reducing hematoma volume (Figure 3B), followed by tranexamic acid (MD: −5.34, 95% CrI: −15.77–4.92, Figure 3B), perindopril (MD: 0.52, 95% CrI: −27.86–28.76, Figure 3B), and goreisan (MD: 6.63, 95% CrI: −2.45–15.70, Figure 3B); the ranking positions of each drug are presented in Figure 4B. Four eligible trials totaling 1,171 patients provided data on a good recovery, the results of which are shown in Figure 3C. There was no significant difference in the good recovery between atorvastatin and dexamethasone (RR: 1.58, 95% CrI: 0.82–2.96). Atorvastatin (RR: 1.64, 95% CrI: 0.92–2.89, Figure 3C) had the highest probability of being ranked first for a good recovery, followed by dexamethasone (RR: 1.03, 95% CrI: 0.79–1.40, Figure 3C). The ranking positions of each drug are presented in Figure 4C. League tables of the efficacy outcomes are presented in Supplementary Table 3. The results remained robust in the sensitivity analysis (Supplementary Table 4).

### Safety Outcome

As regards all-cause mortality, data were only available for two treatment regimens involving 1,191 patients. Our analysis demonstrated that dexamethasone increased the risk of all-cause mortality (RR: 1.96, 95% CrI: 1.20–3.28, Table 2). Atorvastatin was not associated with increased risk of mortality (RR: 2.36, 95% CrI: 0.19–72.22, Table 2). The ranking probabilities graph showed that atorvastatin had the highest probability of being ranked first, followed by dexamethasone (Supplementary Figure 3). We also extracted adverse events in the intervention group reported by each trial, the data of which are shown in Table 3. The majority of adverse reactions were mild and did not require further therapy. However, the adverse events resulting from dexamethasone treatment were generally serious, and even fatal in some cases. League table of the all-cause mortality is presented in Supplementary Table 3.

**Table 2:** Network meta-analysis of all-cause mortality and cause of death.

**Intervention**	**Trial**	**Network RR of mortality compared with placebo and cause of death**
Perindopril		**NA**
	Poulsen et al., 2014	—
Atorvastatin		**2.36 [0.19, 72.22]**
	Jiang et al., 2018	I: Pulmonary embolism (1)
		C: Myocardial infarction (1)
	Yang et al., 2020	—
Goreisan		**NA**
	Katayama et al., 2018	—
	Yamada and Natori, 2020	—
	Fujisawa et al., 2021	—
Tranexamic acid		**NA**
	Workewych et al., 2018	—
	Wan et al., 2020	—
	Yamada and Natori, 2020	—
Dexamethasone		**1.96 [1.20, 3.28]**
	Chan et al., 2015	I: Chest infection and subdural empyema (3)
		C: Intracranial hemorrhage and chest infection (3)
	Prud'homme et al., 2016	I: Possibly severe adverse events of corticosteroid therapy (2)
		C: 0
	Hutchinson et al., 2020	I: 30 deaths
		C: 17 deaths
	Mebberson et al., 2020	I: 4 deaths
		C: 2 deaths

*NA, not available; I, intervention; C, control; RR, relative risk. Number of deaths were presented in the bracket. The RR with CrIs are highlighted in bold prints*.

**Table 3:** Treatment-related adverse events in intervention group.

**Trial**	**Intervention**	**Patient no**.	**Treatment-related adverse events**
Poulsen et al., 2014	Perindopril 5 mg	25	NA
Jiang et al., 2018	Atorvastatin 20 mg	98	Diplopia and right-abduction nerve palsy (1), pruritus (1)
Yang et al., 2020	Atorvastatin 20 mg	28	Abdominal discomfort (3)
Katayama et al., 2018	Goreisan 7.5 g	92	NA
Yamada and Natori, 2020	Goreisan 7.5 g	78	Increased urination frequency (1)
Fujisawa et al., 2021	Goreisan 7.5 g	104	Severe headache (1), diarrhea (1), and abdominal discomfort (1)
Workewych et al., 2018	TXA 500 mg	11	Sinus pain (1), jaw pain (1), dysphagia (1), acute swelling (2), dry skin (1), muscle pain (1), seizure (1), bladder infection (1), and attention and memory impairment (1)
Wan et al., 2020	TXA 1,000 mg	41	Thalamic infarct (1), jaundice (1)
Yamada and Natori, 2020	TXA 750 mg	72	NA
Chan et al., 2015	Dexamethasone 24 mg	122	Chest infection (5), Subdural empyema (1)
Prud'homme et al., 2016	Dexamethasone 12 mg	10	Hyperglycemia (4), hypertension (1), pulmonary embolus (1), cellulitis (1), pulmonary edema (1), suicide (1)
Hutchinson et al., 2020	Dexamethasone 12 mg	375	Adverse events potentially related to dexamethasone (41)
Mebberson et al., 2020	Dexamethasone 12 mg	23	Delirium (3), hyponatremia (1), Pneumonia (1)

*NA: not available. Number of people suffered from adverse events were presented in the bracket*.

### Quality of Evidence Assessment

The quality of evidence for the network comparisons of atorvastatin vs. placebo in recurrence requiring surgery was rated as “high”; dexamethasone vs. placebo in recurrence requiring surgery was rated as “high.” The quality of evidence for the network comparisons of atorvastatin vs. placebo in reducing hematoma volume was rated as “high,” whereas that of atorvastatin vs. goreisan in reducing hematoma volume was rated as “low” due to indirectness and imprecision; tranexamic acid vs. goreisan in reducing hematoma volume was rated as “moderate” due to imprecision. The quality of evidence for the network comparisons of dexamethasone vs. placebo in increasing mortality was rated as “moderate” due to imprecision (Supplementary Table 5).

## Discussion

Atorvastatin, tranexamic acid, and dexamethasone are commonly used drugs for the treatment of CSDH. However, a comprehensive ranking of these treatment regimens with regard to efficacy and safety is lacking. Therefore, we conducted a network meta-analysis to create a comprehensive assessment for ranking the currently available drug therapies for CSDH based on their clinical effects. The present network meta-analysis pooled data derived from 12 RCTs and focused on 5 different drugs involving 2,098 patients. Our results demonstrated that atorvastatin and dexamethasone decreased the incidence of CSDH recurrence requiring surgery compared to placebo. We also found that dexamethasone was significantly superior to goreisan with regard to reducing recurrence. Moreover, atorvastatin showed a better efficacy than the placebo in reducing the hematoma volume. Furthermore, atorvastatin and tranexamic acid reduced the hematoma volume to a greater extent than goreisan. According to the included studies, the adverse events caused by dexamethasone were generally serious, and even fatal in some cases. In line with this, dexamethasone was found to increase the risk of all-cause mortality compared to the placebo. Our findings might be beneficial for clinicians to select the optimal treatment for this group of patients.

We also provided ranking positions of each treatment regimen in different outcomes. However, although rankograms are a direct way to compare the effects of different outcomes in a NMA, the results should be interpreted with caution. In particular, treatment rankings should not be interpreted in isolation, as they do not represent the magnitude of differences between these drugs, but rather indicate the ranking of one of several clinical outcomes.

In the current study, as mortality data were reported in only two drug classes, the safety of other drugs, including tranexamic acid, goreisan, and perindopril, remains uncertain. Furthermore, as only one study of atorvastatin reported the mortality outcome (only two cases unrelated to the treatment), the width of the confidence interval is wide, resulting in an extremely low level of evidence. Moreover, all-cause mortality is a suboptimal indicator of drug safety, as the majority of patients with CSDH are the elderly with multiple comorbid underlying conditions. In this case, we extracted data on cause of death into Table 2 as well as the adverse events of each drug into Table 3. Most of the included studies reported treatment-related adverse reactions. In studies that lacked data on drug-related adverse reactions, we extracted the adverse reactions that were more frequently reported in the treatment group to be used in our study. These findings will allow clinicians to consider the common side effects of these drugs prior to deciding to whether to use them in a treatment regimen.

With regard to recurrence, there is some inconsistency in the comparison between tranexamic acid and goreisan (Supplementary Table 6). Additionally, as only one trial compared these two drugs, the results were not robust, and therefore, the level of evidence was downgraded to “low.” More studies are needed before drawing definitive conclusions on the relationship between these drugs and recurrence.

### Comparison With Other Studies

To the best of our knowledge, two previous systematic reviews have evaluated the effects of pharmacological treatment on clinical outcomes in patients with CSDH (Edlmann et al., 2020; Scerrati et al., 2020). Although these previous studies summarized completed and currently running RCTs assessing pharmacological agents in patients with CSDH, they did not perform comprehensive literature searches and quantitative analyses. Several meta-analyses have focused on the effects of single drugs in patients with CSDH. In 2017, a meta-analysis of five trials examined the outcomes of patients who received dexamethasone for CSDH (Yao et al., 2017), and showed that dexamethasone, alone or as an adjuvant to surgery, resulted in a lower recurrence rate (RR, 0.54; 95% CI, 0.33–0.88; *I*^2^ = 43%). In 2019, Holl et al. reported that the addition of corticosteroids to surgery led to lower all-cause mortality than surgery alone (RR, 0.39; 95% CI, 0.25–0.63; *I*^2^ =15%) (Holl et al., 2019). These results are in contrast to our findings. However, these previous studies mostly included retrospective studies, with the exception of one trial, which led to significant bias. We also conducted direct comparisons with regard to all-cause mortality, the results of which remained consistent with the network comparisons (Supplementary Figure 4). Moreover, a previous systematic review discussed the effect of atorvastatin in the context of CSDH, although they did not perform quantitative analysis due to data limitations (Qiu et al., 2017).

In contrast, the methodology and data used in the current study are different from those used in previous meta-analyses. First, we performed a network meta-analysis, which is a more powerful method to provide a comprehensive analysis of evidence, especially in the absence of head-to-head research. Second, unlike previous systematic reviews and meta-analyses, we only included RCTs that assessed different drugs for patients with CSDH, therefore avoiding selection bias. Moreover, we used the GRADE approach to rate the quality of evidence. Finally, our analysis enriched previously published meta-analyses as we included all available trials from previous meta-analyses and additional trials identified by systematic database searching.

### Study Implications

Currently, high-level guidelines to guide CSDH treatment are lacking. One study published in 2017 summarized studies that evaluated the treatment of CSDH with various conservative treatment modalities. The findings suggested that all of the drug treatment options for CSDH should be considered grade C recommendations due to restrictions regarding the available data (Soleman et al., 2017); these recommendations were mostly based on the findings of previous retrospective studies and small RCTs (Berghauser Pont et al., 2012a). In the current study, we found high quality evidence that atorvastatin and dexamethasone decreased recurrence in patients with CSDH. Moreover, high quality evidence suggested that atorvastatin reduced the hematoma volume compared with placebo. The present meta-analysis should spur the addition of drug treatment for CSDH in future guidelines.

The ideal pharmacological treatment of CSDH should reduce recurrence and improve functional recovery while also decreasing hematoma volume, side effects, and mortality. Based on the present analysis, atorvastatin seems to be the optimal treatment choice. Moreover, animal models have shown that atorvastatin improves neurological function in patients with CSDH by reducing inflammation-induced vascular leakage, promoting angiogenesis, preventing hematoma formation, and accelerating hematoma resorption (Quan et al., 2015; Wang et al., 2016). Although dexamethasone can reduce the recurrence rate, almost all of the available trials found that dexamethasone was associated with more adverse events than the placebo. Although most of adverse events were mild and did not require further treatment, it is important to consider that dexamethasone can also cause severe adverse effects, including hyperglycemia, thrombosis, and infections, which can even lead to death in some cases.

### Limitations

This meta-analysis has several limitations. First, the inclusion of open-label trials led to both performance and detection bias. Second, although most of the drugs were studied as adjuvant treatments to surgery, others were designed to replace surgery. We excluded trials that used drugs as an alternative to surgery as sensitivity analysis, and the results remained consistent (Supplementary Table 4). Third, owing to insufficient data on endpoints such as death in the included studies, it was difficult to accurately determine the safety of most drugs in this way. Therefore, we further extracted data on treatment-related adverse events of each drug in the Table 3. Fourth, there were differences across the included studies with regard to treatment (frequency and duration of medication; whether or not the patients underwent pre-operation), outcome (definition of recurrence), and follow-up. This diversity may have influenced the effect of drug therapy on patients with CSDH.

## Conclusion

Our analysis suggest that dexamethasone is the best treatment to reduce recurrence and atorvastatin is the best treatment to reduce hematoma volume in patients with CSDH. Although dexamethasone can reduce the recurrence, clinicians should pay close attention to the elevated risk of all-cause mortality and potential adverse events. Future well-designed RCTs with more participants are needed to verify these findings.

## Data Availability Statement

The original contributions presented in the study are included in the article/Supplementary Material, further inquiries can be directed to the corresponding author/s.

## Author Contributions

XW and CY designed the meta-analysis. XW and QH searched for relevant studies, selected the studies, and extracted the relevant information. XW and JS synthesized the data. XW wrote the first draft of the paper. All authors revised the manuscript and approved the final manuscript as submitted and agree to be accountable for all aspects of the work.

## Conflict of Interest

The authors declare that the research was conducted in the absence of any commercial or financial relationships that could be construed as a potential conflict of interest.
