# Supplementary material for: Pharmacological Treatment in the Management of Chronic Subdural Hematoma
Source: Front Aging Neurosci. 2021 Jul 1;13:684501. doi: 10.3389/fnagi.2021.684501 (PMC8280518; doi:10.3389/fnagi.2021.684501)
Supplement: Supplementary file 1 [file Table_1.DOCX]

### Table S1: Search Strategy

| OVID EMBASE (adapted for other databases) | |
| --- | --- |
| 1 | exp Hematoma, Subdural, Chronic/ |
| 2 | (Chronic Subdural Hematoma* OR Subdural Hematoma*, Chronic OR Hematoma*, Chronic Subdural OR Chronic Subdural Haematoma* OR Subdural Haematoma*, Chronic OR Haematoma*, Chronic Subdural).ab,kw,ti |
| 3 | 1 or 2 |
| 4 | exp Drug Therapy/ OR (Pharmacotherap*).ab,kw,ti |
| 5 | exp Conservative Treatment/ OR (Conservative Management*).ab,kw,ti |
| 6 | exp Tranexamic Acid/ OR (Transamin or Anvitoff or AMCA or Amchafibrin or Ugurol).ab,kw,ti |
| 7 | exp Dexamethasone/ OR (Methylfluorprednisolone or Dexasone).ab,kw,ti |
| 8 | exp Atorvastatin/ OR (Liptonorm OR CI 981).ab,kw,ti |
| 9 | exp Angiotensin-Converting Enzyme Inhibitors/ OR (Perindopril or Celecoxib or Goreisan).ab,kw,ti |
| 10 | 4 or 5 or 6 or 7 or 8 or 9 |
| 11 | randomized controlled trial.pt. |
| 12 | controlled clinical trial.pt. |
| 13 | (randomly OR randomized OR placebo OR trial).ab,kw,ti |
| 14 | 6 or 7 or 8 or 9 |
| 15 | exp animals/ |
| 16 | exp humans/ |
| 17 | 15 not 16 |
| 18 | 14 not 17 |
| 19 | 3 and 10 and 18 |
