# Supplementary material for: Pharmacological Treatment in the Management of Chronic Subdural Hematoma
Source: Front Aging Neurosci. 2021 Jul 1;13:684501. doi: 10.3389/fnagi.2021.684501 (PMC8280518; doi:10.3389/fnagi.2021.684501)
Supplement: Supplementary file 2 [file Table_2.DOCX]

Supplemental Table S2. Additional Statistical Methods

| Outcomes | NMA models | DIC | Global heterogeneity | |
| --- | --- | --- | --- | --- |
|  |  |  | Pair-wise analysis | Network analysis |
| Recurrence | Random effect model | 40.43 |  |  |
|  | Fixed effect model (used) | 39.68 | 0 | 0 |
| Changes in HV | Random effect model | 20.45 |  |  |
|  | Fixed effect model (used) | 20.02 | 0 | 0 |
| Good recovery | Random effect model (used) | 15.09 | 60.48% | 60.78% |
|  | Fixed effect model | 16.58 |  |  |
| All-cause mortality | Random effect model | 16.98 |  |  |
|  | Fixed effect model (used) | 15.96 | 0 | 0 |

DIC: Deviance Information Criteria

HV: Hematoma Volume

NMA: Network Meta-Analysis
