# Supplementary material for: Pharmacological Treatment in the Management of Chronic Subdural Hematoma
Source: Front Aging Neurosci. 2021 Jul 1;13:684501. doi: 10.3389/fnagi.2021.684501 (PMC8280518; doi:10.3389/fnagi.2021.684501)
Supplement: Supplementary file 3 [file Table_3.DOCX]

Supplemental Table S3: League tables of outcomes

1. Recurrence required for surgery

| Atorvastatin |  |  |  |  |
| --- | --- | --- | --- | --- |
| 1.19 (0.54, 2.60) | Dexamethasone |  |  |  |
| 0.55 (0.25, 1.19) | 0.46 (0.23, 0.94) | Goreisan |  |  |
| 0.95 (0.35, 2.84) | 0.8 (0.31, 2.29) | 1.73 (0.72, 4.66) | Tranexamic acid |  |
| 0.45 (0.24, 0.81) | 0.38 (0.22, 0.63) | 0.82 (0.49, 1.36) | 0.48 (0.19, 1.04) | Placebo |

1. Changes in hematoma volume

| Atorvastatin |  |  |  |  |
| --- | --- | --- | --- | --- |
| -14.09 (-23.35, -4.82) | Goreisan |  |  |  |
| -7.99 (-36.34, 20.40) | 6.05 (-23.34, 35.73) | Perindopril |  |  |
| -2.07 (-12.59, 8.51) | 12.07 (2.29, 21.68) | 6.00 (-24.23, 36.21) | Tranexamic acid |  |
| -7.44 (-9.49, -5.43) | 6.63 (-2.45, 15.70) | 0.52 (-27.86, 28.76) | -5.34 (-15.77, 4.92) | Placebo |

1. Good recovery

| Atorvastatin |  |  |
| --- | --- | --- |
| 1.58 (0.82, 2.96) | Dexamethasone |  |
| 1.64 (0.92, 2.89) | 1.03 (0.79, 1.39) | Placebo |

1. All-cause mortality

| Atorvastatin |  |  |
| --- | --- | --- |
| 1.20 (0.09, 38.48) | Dexamethasone |  |
| 2.36 (0.19, 72.22) | 1.96 (1.21, 3.28) | Placebo |
