# Supplementary material for: Pharmacological Treatment in the Management of Chronic Subdural Hematoma
Source: Front Aging Neurosci. 2021 Jul 1;13:684501. doi: 10.3389/fnagi.2021.684501 (PMC8280518; doi:10.3389/fnagi.2021.684501)
Supplement: Supplementary file 4 [file Table_4.DOCX]

Supplementary Table S4. Sensitivity analysis for the outcomes.

1. Recurrence required for operation (RR with 95 CrIs)

| Atorvastatin |  |  |  |  |
| --- | --- | --- | --- | --- |
| 0.68 (0.09, 3.00) | Dexamethasone |  |  |  |
| 0.3 (0.04, 1.27) | **0.44 (0.21, 0.91)** | Goreisan |  |  |
| 0.52 (0.06, 2.65) | 0.76 (0.29, 2.17) | 1.73 (0.71, 4.66) | Tranexamic acid |  |
| **0.25 (0.03, 0.97)** | **0.36 (0.21, 0.61)** | 0.83 (0.5, 1.36) | 0.48 (0.19, 1.04) | Placebo |

1. Change in hematoma volume (MD with 95 CrIs)

| Atorvastatin |  |  |  |  |
| --- | --- | --- | --- | --- |
| **-13.71 (-23.14-, 4.36)** | Goreisan |  |  |  |
| -7.77 (-36.09, 20.47) | 6.09 (-23.72, 35.42) | Perindopril |  |  |
| -1.72 (-12.37, 8.71) | **11.96 (2.26, 21.64)** | 5.99 (-23.77, 36.03) | Tranexamic acid |  |
| **-7.10 (-9.22, -5.03)** | 6.60 (-2.43, 15.67) | 0.66 (-27.64, 28.89) | -5.38 (-15.58, 5.05) | Placebo |

MD: mean difference; RR: relative risk; CrI: credibility interval
