# Supplementary material for: Pharmacological Treatment in the Management of Chronic Subdural Hematoma
Source: Front Aging Neurosci. 2021 Jul 1;13:684501. doi: 10.3389/fnagi.2021.684501 (PMC8280518; doi:10.3389/fnagi.2021.684501)
Supplement: Supplementary file 5 [file Table_5.DOCX]

| Outcomes | Direct comparison | Direct Estimate (95% CrI) | Indirect Estimate  (95% CrI) | Inconsistency P value |
| --- | --- | --- | --- | --- |
| Recurrence | TXA vs Goreisan | 0.10 (0.01, 0.62) | 1.45 (0.42, 4.80) | 0.016 |

Supplemental Table S5: Node-splitting model results for the outcomes.
