# Supplementary material for: Pharmacological Treatment in the Management of Chronic Subdural Hematoma
Source: Front Aging Neurosci. 2021 Jul 1;13:684501. doi: 10.3389/fnagi.2021.684501 (PMC8280518; doi:10.3389/fnagi.2021.684501)
Supplement: Supplementary file 6 [file Table_6.DOCX]

Supplemental Table S6: GRADE summary for the outcomes

1. Recurrence required for surgery

| Atorvastatin |  |  |  |  |
| --- | --- | --- | --- | --- |
| Low ^#§^ | Dexamethasone |  |  |  |
| Low ^#§^ | Low ^#§^ | Goreisan |  |  |
| Low ^#§^ | Low ^#§^ | Low ^#&^ | Tranexamic acid |  |
| High | High | Moderate ^#^ | Moderate ^#^ | Placebo |

1. Changes in hematoma volume

| Atorvastatin |  |  |  |  |
| --- | --- | --- | --- | --- |
| Low ^#§^ | Goreisan |  |  |  |
| Very low *^§^ | Very low *^§^ | Perindopril |  |  |
| Low ^#§^ | Moderate ^#^ | Very low *^§^ | Tranexamic acid |  |
| High | Moderate ^#^ | Low * | Moderate ^#^ | Placebo |

1. Good recovery

| Atorvastatin |  |  |
| --- | --- | --- |
| Low ^#§^ | Dexamethasone |  |
| Moderate ^#^ | Moderate ^#^ | Placebo |

1. All-cause mortality

| Atorvastatin |  |  |
| --- | --- | --- |
| Very low *^§^ | Dexamethasone |  |
| Low * | Moderate ^#^ | Placebo |

^#^ serious imprecision

*very serious imprecision

^§^ serious indirection

^&^ serious inconsistency
