# Supplementary material for: Pharmacological Treatment in the Management of Chronic Subdural Hematoma
Source: Front Aging Neurosci. 2021 Jul 1;13:684501. doi: 10.3389/fnagi.2021.684501 (PMC8280518; doi:10.3389/fnagi.2021.684501)
Supplement: Supplementary file 7 [file Data_Sheet_1.docx]

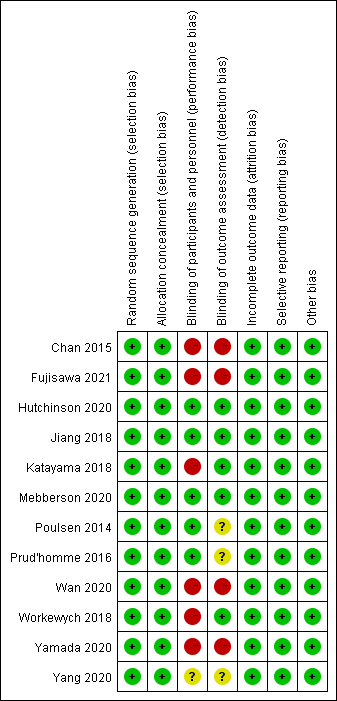


**Supplementary Figure S1.** Risk of bias summary of included trials.


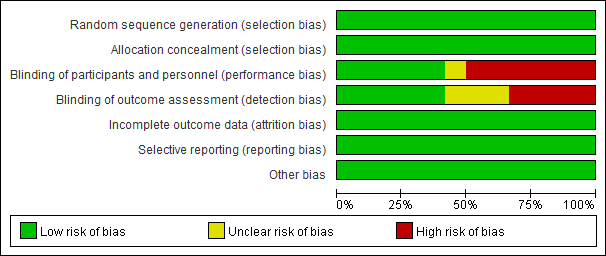


**Supplementary Figure S2.** Risk of bias graph of included trials.


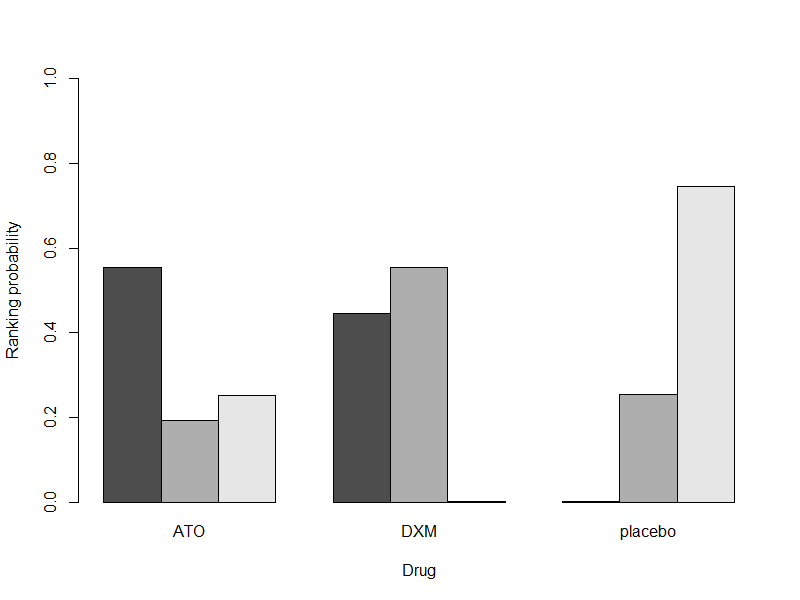


Supplementary Figure S3. Ranking probabilities graph of each medication for all-cause mortality


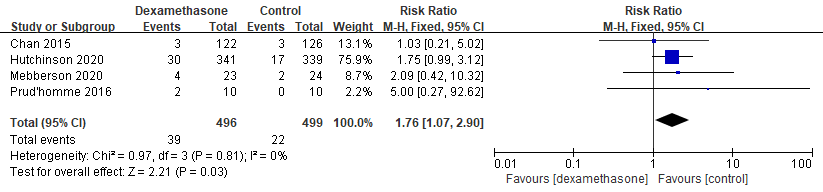


**Supplementary Figure S4.** Direct meta-analysis for all-cause mortality of dexamethasone.
